# From localization to function: comparative analysis of CB1 in sperm across species and its epigenetic role in humans

**DOI:** 10.1038/s41419-025-08386-2

**Published:** 2026-01-15

**Authors:** Marta Lombó, Fiorenza Sella, Christian Giommi, Stefano Giannubilo, Andrea Frontini, Andrea Ciavattini, Gilda Cobellis, Franceso Manfrevola, Nina Montik, Marina Paolanti, Paz Herráez, Oliana Carnevali

**Affiliations:** 1 Department of Life and Environmental Sciences, Università Politecnica delle Marche, Ancona, Italy; 2 Department of Molecular Biology, Faculty of Biology and Environmental Sciences, Universidad de León, León, Spain; 3 INBB—Consorzio Interuniversitario di Biosistemi e Biostrutture, Roma, Italy; 4 Department of Odontostomatological and Specialized Clinical Sciences, Università Politecnica delle Marche, Ancona, Italy; 5 Department of Experimental Medicine, University of Campania L. Vanvitelli, Naples, Italy; 6 Department of Political Sciences, Communication and International Relations, University of Macerata, Macerata, Italy

**Keywords:** Infertility, Super-resolution microscopy

## Abstract

The endocannabinoid system (ECS) is evolutionarily conserved and regulates key physiological processes, including sperm motility and capacitation. However, the localization and function of cannabinoid receptor 1 (CB1) in human sperm remain debated, with prior widefield microscopy studies producing inconsistent results. Using confocal and Airyscan microscopy, we mapped CB1 distribution in human sperm, revealing a dotted pattern along the tail, presence in some midpieces, and discrete spots in the head. Additionally, a comparative study revealed that CB1 was present in the sperm tail of invertebrates and vertebrates, while it was only detected in the sperm head of roosters (restricted to the acrosomal region) and mammals. Notably in mammalian sperm, a subset of CB1 receptors was detected intracellularly, beneath the plasma and outer acrosomal membranes, extending toward the nuclear region, where it persisted even after the acrosome reaction. These data support additional role beyond sperm motility and capacitation-induced acrosome reaction. Given that CB1 is involved in chromatin remodeling in murine sperm, we investigated whether it plays a similar role in human sperm. Our findings demonstrate that CB1 activation by the specific agonist Arachidonyl-2’-chloroethylamide (ACEA) enhances histone H4 acetylation, restoring levels in asthenoteratozoospermic samples to those of normozoospermic donors. Interestingly, while N-arachidonoylethanolamine (AEA) treatment reduced sperm DNA fragmentation, ACEA had no such effect, evidencing that DNA fragmentation is not CB1-mediated. As established in mammals, the histone-to-protamine transition is a critical phase of chromatin remodeling and our study highlights a conserved role for CB1 in regulating chromatin dynamics during this process.

## Introduction

Gametogenesis is a complex process requiring precise regulation by the hypothalamic-pituitary-gonadal axis. However, more recently, additional modulators of this reproductive process have been identified, including lipid-derived bioregulators collectively known as endocannabinoids [1]. These ligands, such as Anandamide (AEA) and 2-Arachidonoylglycerol, form the endocannabinoid system (ECS) along with their biosynthetic and degrading enzymes, as well as canonical (cannabinoid receptor 1 and 2, CB1 and CB2) and non-canonical receptors (such as the channels transient receptor potential vanilloid) [2]. The involvement of the ECS in male reproductive events was first recognized when Schuel et al. [3] demonstrated that AEA treatment reduced sea urchin (*Strongylocentrotus purpuratus*) sperm fertilizing capacity. Subsequent studies found similar results in human sperm, showing that treatment with R-methanandamide, a potent and metabolically stable AEA analog, also reduced sperm fertilizing ability by 50% [4]. In the same study, the authors reported that relatively high AEA concentrations in human seminal plasma ( ~ 12 nM) likely prevent premature capacitation in newly ejaculated spermatozoa, while its levels gradually decrease throughout the female reproductive tract, facilitating capacitation and fertilization [4]. A later study showed that incubation of sperm with exogenous AEA decreased sperm motility by impairing mitochondrial function and inhibited capacitation-induced acrosome reaction through CB1 activation [5].

CB1 is one of the most abundant G protein-coupled receptors (GPCRs) in the central nervous system, localized not only at the cell surface but also intracellularly [6]. Recent evidence challenges the traditional view that GPCRs signal exclusively from the plasma membrane. Instead, numerous GPCRs have been shown to localize to endomembrane compartments, where they initiate distinct signaling cascades [7]. Particularly, upon ligand activation, CB1 signals in three distinct spatiotemporal waves: an initial transient response mediated by heterotrimeric G proteins, followed by a second wave involving β-arrestins, and a final intracellular wave triggered by either G proteins or β-arrestins, each with its own downstream effectors [8]. The presence and function of this receptor appear to be conserved not only in the central nervous system but also in the gonads, as reviewed by Battista et al. [9]. Moreover, CB1 and/or its encoding gene, *CNR1*, have been detected in the testes and sperm of various invertebrate and vertebrate species [1]. In sea urchin sperm, approximately 700 cannabinoid receptors per cell have been identified, and their activation inhibits the egg jelly-stimulated acrosome reaction [10]. In frogs (*Rana esculenta*), CB1 has been detected in both the testicular germinal compartment and isolated spermatozoa, where its activation reduces sperm motility [11]. In fish, the CB1-like receptor gene was identified in the gonads of gilthead seabream (*Sparus aurata*), a species that undergoes sex reversal, with higher expression levels in both mature testes and sex-reversing gonads compared to the mature ovary [12]. The gene encoding CB1, *cnr1*, has also been detected in the testes and ovaries of zebrafish (*Danio rerio*) [13, 14], as well as in goldfish (*Carassius auratus*) and *Pelvicachromis pulcher* [14]. In mammals, CB1 has been detected through immunocytochemical analysis in both the postacrosomal and middle regions of boar sperm cells, where its activation inhibits the acrosome reaction via a cAMP-dependent pathway [15]. In bulls, CB1 is localized on the sperm head, specifically in the equatorial segment and acrosomal region, playing a role in sperm capacitation and release from the bovine oviductal epithelium [16, 17]. Activation of both CB1 and CB2 receptors has been shown to enhance cAMP activity and mitogen-associated protein kinase signaling, leading to bull sperm hyperactivation and acrosome reaction, while blocking these receptors significantly reduces progesterone-induced capacitation and acrosome reaction [18]. In rodents, Wenger et al. [19] were the first to demonstrate the presence of CB1 in Leydig cells and its direct role in regulating luteinizing hormone and testosterone secretion using *Cnr1*^*−/−*^ male mice. The expression of *Cnr1* in the testis varies throughout postnatal development in mice, showing high levels at one week postpartum, decreasing during the prepubertal stage, and peaking in adulthood. CB1 is found in different cells at different stages of sperm development, being initially present in spermatogonia, primary spermatocytes, and Leydig cells, before becoming strongly expressed in Leydig cells, seminiferous tubule epithelia, and spermatozoa in adult testes [20]. Western blot analysis confirmed the presence of CB1 protein in both mice and rat sperm lysates [11]. Additionally, immunohistochemical analysis of developing rat testes revealed CB1 expression in somatic cells, including Leydig and Sertoli cells, as well as in round and elongating spermatids and spermatozoa. In rat sperm cells, CB1 was specifically localized along the head and middle piece membranes [21]. Beyond its role in steroidogenesis and Leydig cell differentiation, studies using *Cnr1*-null mice have demonstrated its crucial function in preventing premature sperm motility within the epididymis [21] and in regulating chromatin remodeling in spermatids by increasing transition protein 2 levels and enhancing histone displacement [22]. More recently, it was shown that *Cnr1*-gene deletion disrupts not only mice histone displacement in spermatids by regulating the hyperacetylation of histone H4 in the testes, but also sperm chromatin condensation by impairing protamine disulfide bond formation in the epididymis [23].

In humans, *CNR1* mRNA and CB1 protein have been detected in sperm and testis by multiple authors [5, 24–26]. However, discrepancies exist regarding CB1 localization in human sperm cells. The first study by Rossato et al. [5] used immunofluorescence to identify CB1 in the head and midpiece, with little to no staining in the tail. Their findings showed that CB1 activation by AEA reduces sperm motility and inhibits the capacitation-induced acrosome reaction. Similarly, Francavilla et al. [27] demonstrated, via immunocytochemistry, that CB1 is restricted to the postacrosomal region and midpiece. In contrast, Agirregoitia et al. [26] reported CB1 presence not only in the plasma membrane of the sperm head (with around 15% of cells exhibiting prolonged staining in the postacrosomal region) and midpiece, but also along the tail. Functionally, incubation with the selective CB1 agonist arachidonyl-2’-chloroethylamide (ACEA) increased the proportion of immotile sperm, supporting the results of Rossato and colleagues [5]. Immunogold labeling further revealed CB1 compartmentalization at the sperm head membranes and midpiece, primarily in mitochondria, with reduced labeling in the tail [25]. In this study, Aquila and colleagues proposed CB1 as a regulator of sperm survival via PI3K/Akt pathway and acquisition of fertilizing ability [25]. Noteworthy, in adult human testes, CB1 immunohistochemistry showed cytoplasmic localization in Leydig cells, while in germ cells, particularly primary spermatocytes, it was detected in the nucleus, suggesting a potential nuclear role in germ cell function [24].

In this context, the present study aimed to: (i) map the distribution of CB1 receptor on human spermatozoa, with a particular focus on the sperm head, using high-resolution confocal microscopy; (ii) determine whether CB1 sperm localization is evolutionarily conserved across mammalian and non-mammalian species with different patterns of chromatin compaction; and (iii) investigate the potential role of CB1 on sperm chromatin focusing on DNA integrity and histone H4 acetylation.

## Materials and methods

### Human sperm collection

Human sperm samples (*Homo sapiens sapiens*) were obtained from young donors (20–35 years old) via masturbation following 3–5 days of sexual abstinence. Samples were collected in sterile containers at the Fertility Clinic Centro Medicina della Riproduzione e Tecniche di Fecondazione Assistita (Ospedali Riuniti Ancona, Italy) and the Laboratorio Analisi AB (Ancona, Italy). Ejaculates were maintained at 37 °C and processed within 1 h of collection after liquefaction. Routine seminal parameters were evaluated according to the World Health Organization [28] guidelines, following standards approved by the Territorial Ethical Committee (263/3932/2024). All donors provided informed consent.

### Rat and mice sperm collection

Rat (*Rattus norvegicus*) sperm was kindly provided by the animal facilities of the *Istituto Nazionale Ricovero e Cura Anziani Polo Scientifico Tecnologico* (I.N.R.C.A. P.S.T., Ancona, Italy), while mouse (*Mus musculus*) sperm from caput and cauda epididymis were collected by the Animal Research and Welfare Service of Universidad de León (León, Spain). For sperm collection, animals were euthanized in accordance with the ethical protocols of each center. The deferens ducts, cauda epididymides, and testes were excised with fine scissors and placed in a Petri dish containing PBS to allow sperm swim-out at 37 °C.

### Bull and ram sperm collection

Bull (*Bos taurus*) and ram (*Ovis aries*) samples were kindly provided by the Animal Selection and Reproduction Center of Junta de Castilla y León (CENSYRA, León, Spain). Animals were housed and the standard conditions and routine veterinary inspections were performed to monitor reproductive health and nutrition of these animals. Semen was obtained from two trained males during the breeding season using an artificial vagina, and their ejaculates were pooled.

### Sea urchin sperm collection

Adult sea urchins (*Paracentrotus lividus*) were collected along the Adriatic coast (Ancona, Italy). They were transported to the laboratory in a suitable container partially filled with seawater from the sampling site. Sperm collection was performed by injecting 1 ml of 0.55 M KCl into the coelom through the peristome of three male sea urchins. The collected sperm was transferred to a tube, and aliquots were prepared in seawater for further analysis.

### Zebrafish sperm collection

Zebrafish (*Danio rerio*) sperm collection was performed by ventral squeezing, following the procedure described by González-Rojo and colleagues [29] with minor modifications, once the fish were anesthetized with 168 mg/L ethyl 3-aminobenzoate methanesulfonate (MS222). Sperm from three males was pooled for immunocytochemical analysis. Animals were maintained under the standard conditions (28.0 ± 0.5 °C and 14/10 h of light/dark photoperiod) and fed twice a day with dry food in accordance with the Italian legislation at the Marche Polytechnic University (Ancona, Italy).

### Rooster sperm collection

Sperm was collected from two Leghorn roosters (*Gallus gallus domesticus*) at the Faculty of Veterinary (University of Matellica, Italy) by dorso-abdominal massage in 1.5 mL tubes as indicated by Akhlaghi et al. [30].

### CB1 Immunocytochemistry

For all the species, sperm were fixed in 4% paraformaldehyde (PFA) for 20 min at room temperature (RT). Fixed sperm were washed in deionized water and diluted to a final and concentration of 10^6^ cells/ml, smeared onto slides, and air-dried.

Immunocytochemistry was performed following the protocol described by González-Rojo and colleagues [31]. To detect the CB1 receptor, slides were incubated overnight at 4 °C in a humidified chamber with rabbit monoclonal anti-CB1 antibody (ab259323, Abcam) diluted 1:200 in blocking solution. This was followed by three washes with PBS and a 1-h incubation at 37 °C with Goat Anti-Rabbit IgG H&L (Alexa Fluor® 488) (ab150077, Abcam) at 1:500 dilution in PBS in a humidified chamber. For acrosome staining, mammalian samples (human, rat, mouse, bull, and ram) were incubated with 20 µg/ml Alexa Fluor™ 568-conjugated Lectin PNA (from peanut) for 15 min in a humidified chamber at 37 °C. Nuclei were counterstained with DAPI using Fluoroshield mounting medium (ab104139, Abcam).

Although the exact immunogen used to generate the anti-CB1 antibody (ab259323) is proprietary to Abcam, the company confirmed that it lies within amino acids 300–400 of the human CNR1 sequence (P21554). Sequence alignment of the immunogen used to raise antibody showed 100% identity with the corresponding region in mouse, rat, bull, and ram; 85.71% identity in rooster; and 75% in zebrafish. No CNR1 sequence information is currently available for sea urchin. To increase the robustness of the data, another polyclonal antibody directed against the human CNR1 C-terminal region (aa 450 to C-terminus) was used (ab23703) diluted 1:100 in blocking.

To assess the retention of CB1 receptor in the sperm head post-acrosome reaction, human, mouse, bull, and ram sperm (10^7^ cells/ml) were treated with 10 µM ionophore A23187 for 2 h at 37 °C. Following incubation, the cells were fixed at t0 and after 2 h incubation (t1) and CB1 immunocytochemistry was carried out as previously described.

### In vitro treatment of human sperm with cannabinoids

Human sperm from donors was treated with AEA (A0580, Sigma-Aldrich) and ACEA (A9719, Sigma-Aldrich) to investigate the role of CB1 in sperm nucleus. AEA is an endogenous cannabinoid that interacts with multiple cannabinoid receptors, while ACEA is a selective exogenous agonist with high affinity for CB1.

For in vitro treatments, semen samples were centrifuged at 1000 × *g* at room temperature (RT) and the seminal plasma was discarded. The sperm cell fraction was then resuspended in ORIGIO® Sperm Wash (CooperSurgical®). After counting the cells, three aliquots containing 30 × 10^6^ spermatozoa/ml were prepared and incubated with either 1% (v/v) DMSO (control), 1 µM AEA, or 1 µM ACEA diluted in G-MOPS™ PLUS (Vitrolife) medium for 1 h at 37 °C. Following the treatments, the cells were fixed in 4% PFA for 20 min and washed three times with deionized water. The cells were then stored at 4 °C for further use.

### Histone 4 acetylation

To determine the possible involvement of CB1 on human sperm H4 acetylation, cells subjected to the cannabinoids in vitro treatments were incubated with a rabbit polyclonal anti-H4K12ac antibody (ab46983, Abcam) at a 1:200 dilution, following the same protocol as used for the CB1 antibody. Secondary antibodies included Goat Anti-Rabbit IgG H&L (ab150077, Abcam) and Goat Anti-Mouse IgG H&L (ab175473, Abcam). Acrosome staining was performed using the same dilutions and conditions as previously described.

### Terminal deoxynucleotidyl transferase dUTP nick end labeling (TUNEL) assay

The potential role of CB1 in human sperm DNA integrity was evaluated using a commercial kit (In Situ Cell Death Detection Kit, Fluorescein, Roche-Mannheim). After in vitro cannabinoid treatments, the manufacturer’s instructions were followed with the slight modifications reported by González-Rojo and colleagues [31].

### Western blot

After liquefaction, semen samples were centrifuged at 1000 × *g* at room temperature (RT), and the seminal plasma was discarded. The sperm cell fraction was resuspended in ORIGIO® Sperm Wash (CooperSurgical®). Following cell counting, three aliquots containing 35 × 10^6^ spermatozoa/mL were centrifuged. To eliminate non-sperm cells, the swim-up technique was performed by carefully adding 0.5 mL of G-MOPS™ PLUS (Vitrolife) medium to the pellet. The samples were incubated at a 45° angle for 1 h at 37 °C. The supernatant was aspirated, and the cells were centrifuged again at 1000 × *g* at RT.

For protein extraction, both the sperm pellets were lysed in a buffer containing 0.125 M Tris-HCl (pH 7.5), 4% (w/v) SDS, 20% (v/v) glycerol, and 10% (v/v) β-mercaptoethanol, supplemented with a 1:10 dilution of Protease Inhibitor Cocktail (Sigma-Aldrich). The lysate was heated to 100 °C for 5 min in a water bath, followed by centrifugation at 12,000 × *g* for 15 min at 4 °C. The supernatant was collected, and the protein concentration was determined using Bradford Reagent (Sigma-Aldrich) according to the manufacturer’s instructions. 15 µg of protein were loaded onto a 12% polyacrylamide gel (SDS-PAGE), and electrophoresis was run at 60 mA for 180 min (Bio-Rad). Proteins were transferred to a nitrocellulose membrane (Invitrogen) using a wet transfer system (Bio-Rad) at 250 mA for 2 h at 4 °C. Membranes were blocked in 5% (w/v) BSA in 0.2% (v/v) Tween 20 in Tris-buffered saline (TBST) for 90 min at RT. They were then incubated overnight at 4 °C with rabbit Anti-Histone H4 (ab31830, Abcam), anti-H4K12ac (ab46983, Abcam), and anti-CB1 (ab259323, Abcam) antibodies, all diluted 1:1000. After three 15-min washes with TBST, the membranes were incubated with 1:2500 anti-Mouse and anti-Rabbit IgG horseradish peroxidase-conjugated secondary antibodies (Sigma-Aldrich) for 1 h at 30 °C. Following another three 15-min washes with TBST, HRP-labeled proteins were visualized using Clarity ECL Substrate (Bio-Rad) on a GS800 densitometer (Bio-Rad).

### Confocal imaging

To analyze CB1 presence in sperm from different species, confocal microscopy images were acquired using a Zeiss LSM800 Confocal Laser Scanning Microscope with 63 × Plan-Apo/1.4 or 100 × Plan-Apo/1.46 oil objectives at RT. Z-stack images were captured, and maximum intensity projections were generated. To achieve higher resolution in 3D dimensions, Airyscan imaging was performed with 63 × magnification and 2 × zoom. Images were processed using ZEN Blue software (Carl Zeiss Microscopy GmbH).

To assess the potential role of CB1 in sperm DNA integrity and H4K12 acetylation, confocal images were acquired using a Nikon A1R confocal microscope using 60 × 1.49 numerical aperture oil objective at RT. Fiji ImageJ Software was used to analyze TUNEL-positive cells and intensity levels of H4K12ac in all human sperm samples. A minimum of 200 cells were analyzed per sample.

### Statistical analysis

Statistical analyses were conducted using GraphPad Prism v8.0.1 (GraphPad Software, Inc., San Diego, CA, USA). Data normality was assessed using the Shapiro–Wilk test, and appropriate statistical tests were applied accordingly. For comparisons among treatments, one-way ANOVA with Tukey’s HSD post-hoc test was used for parametric data, while non-parametric data were analyzed using the Kruskal–Wallis test with Dunn’s post-hoc analysis. For comparisons between normozoospermic and asthenozoospermic samples, an unpaired *t*-test with Welch’s correction was applied for parametric data, and the Mann–Whitney test was used for non-parametric data. Statistical significance was set at *p* < 0.05 for all analyse.

### Large language models

English language revision of the manuscript was performed with the assistance of ChatGPT (version 5.0, OpenAI), used to improve clarity and grammar.

## Results

### CB1 specificity

To address anti-CB1 (ab259323, Abcam) specificity, we performed western blot analysis, following the approach suggested by Esteban for anti-CB1 antibodies [32, 33]. Western blotting was performed on human sperm selected by swim-up displayed a single band corresponding to 53 kDa, even after membrane overexposure (Fig. S1, Supplementary Materials).

### Localization of CB1 in human sperm

**Fig. 1:**
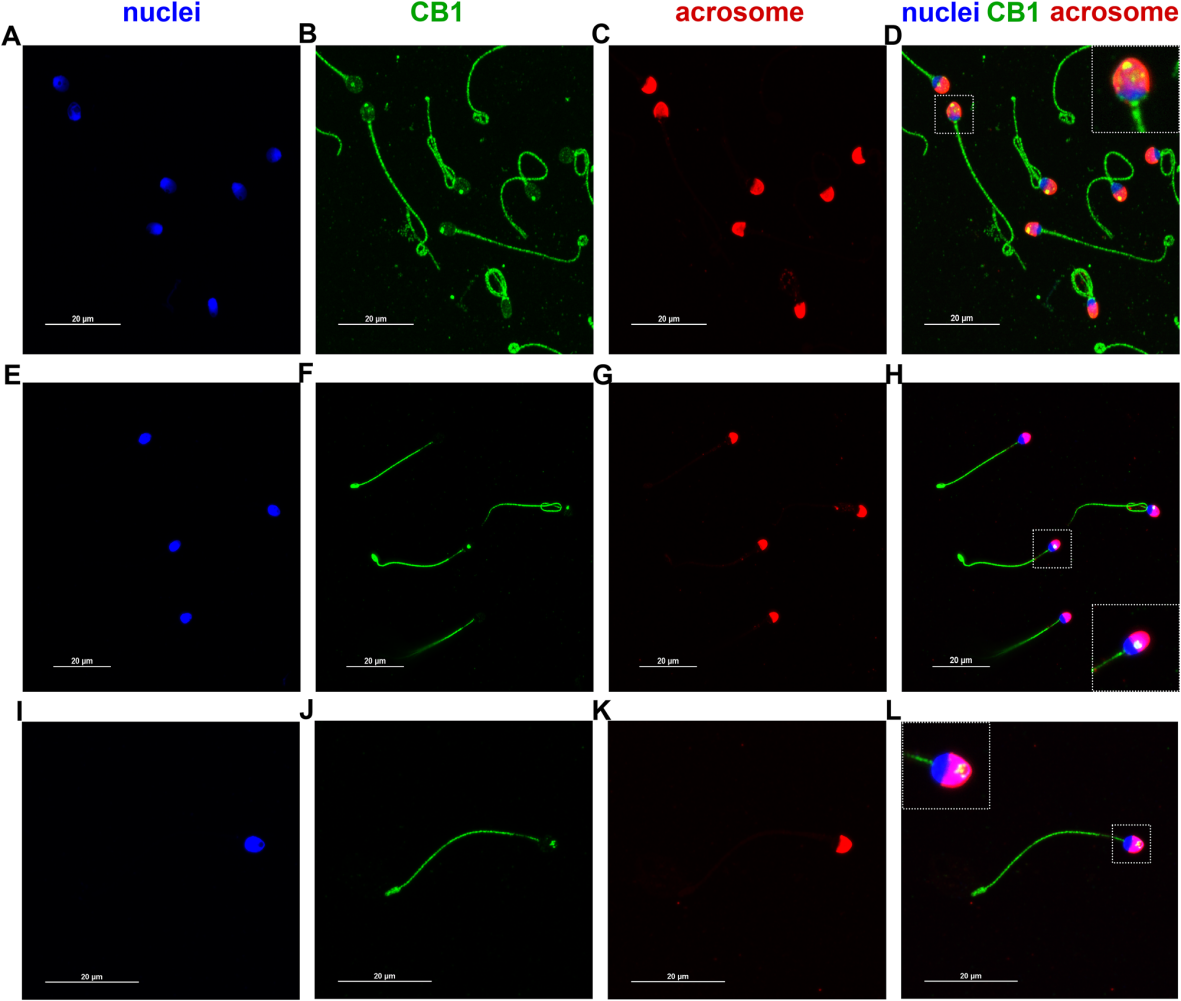
CB1 is present in both the human sperm head and flagellum. Representative confocal images showing CB1 distribution in human sperm, with fluorescence signals (green) detected in the acrosomal (red) and post-acrosomal regions of the head, as well as in a spot-like pattern along the flagellum. Nuclei were counterstained with DAPI (blue). CB1 dots in the acrosomal region appear in yellow, as merge of CB1 (green) and acrosome (red). High-magnification images (white dashed squares) highlight CB1 localization as multiple smaller head spots (**A**–**D**, **I**–**L**) or as a single larger head spot (**E**–**H**). Scale bar: 20 µm.

The immunocytochemical analysis of CB1 in human sperm showed that CB1 exhibited a spot-like distribution along flagellum (Fig. 1A–D), with a weaker signal observed in the midpiece of several spermatozoa (Fig. 1E–L). In addition, CB1 was present in the head of most sperm cells, appearing either as multiple distinct spots in both the acrosomal and post-acrosomal regions (dashed-line enlargements in Fig. 1D, L) or as a single bigger spot in the acrosomal region (dashed-line enlargements in Fig. 1H).

**Fig. 2:**
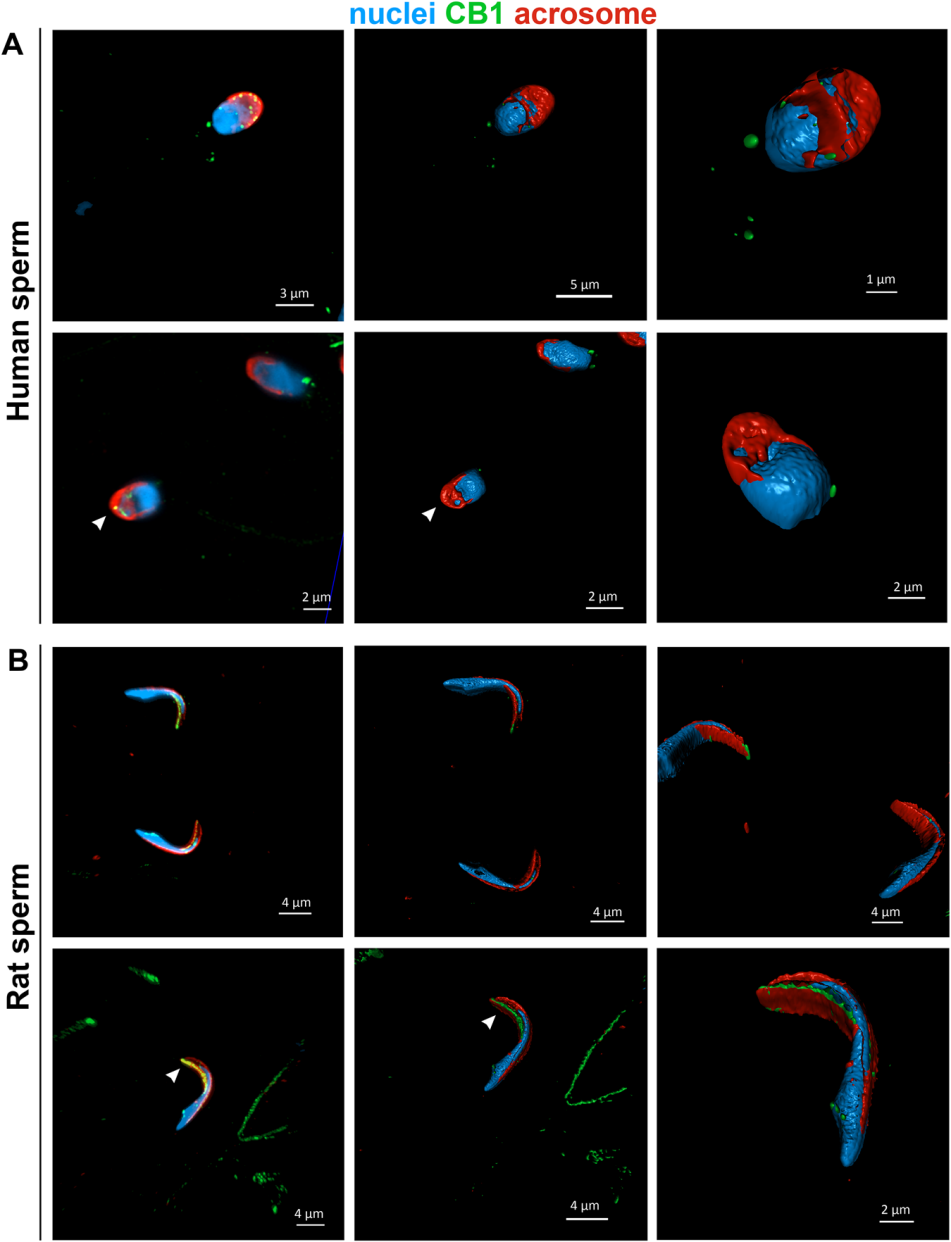
CB1 head spots are localized in the internal part of the acrosome. High-resolution confocal images of human (**A**) and rat (**B**) sperm showing CB1 head spots (green) in human sperm. 3D reconstructions obtained after Airyscan processing confirm that some CB1 head spots (indicated by white arrow heads) are located beneath the outer acrosomal membrane. The acrosome was stained with Alexa Fluor™ 568-conjugated Lectin PNA (red), and nuclei were counterstained with DAPI (blue).

The 3D reconstruction from the z-stack (videos 1 and 2, Supplementary Material) revealed that some CB1 spots in the sperm head are localized below the outer acrosomal membrane. This finding is supported by Airyscan detection, which revealed dispersed CB1 spots in Z-stack sections of both human (Fig. 2A, left) and rat sperm (Fig. 2B, left), predominantly in the acrosomal region extending toward the nuclear region. However, when surface reconstruction was performed (central and right images), most of these CB1 spots were no longer visible, confirming their intracellular localization.

### Localization of CB1 in mammalian sperm head before and after acrosome reaction

**Fig. 3:**
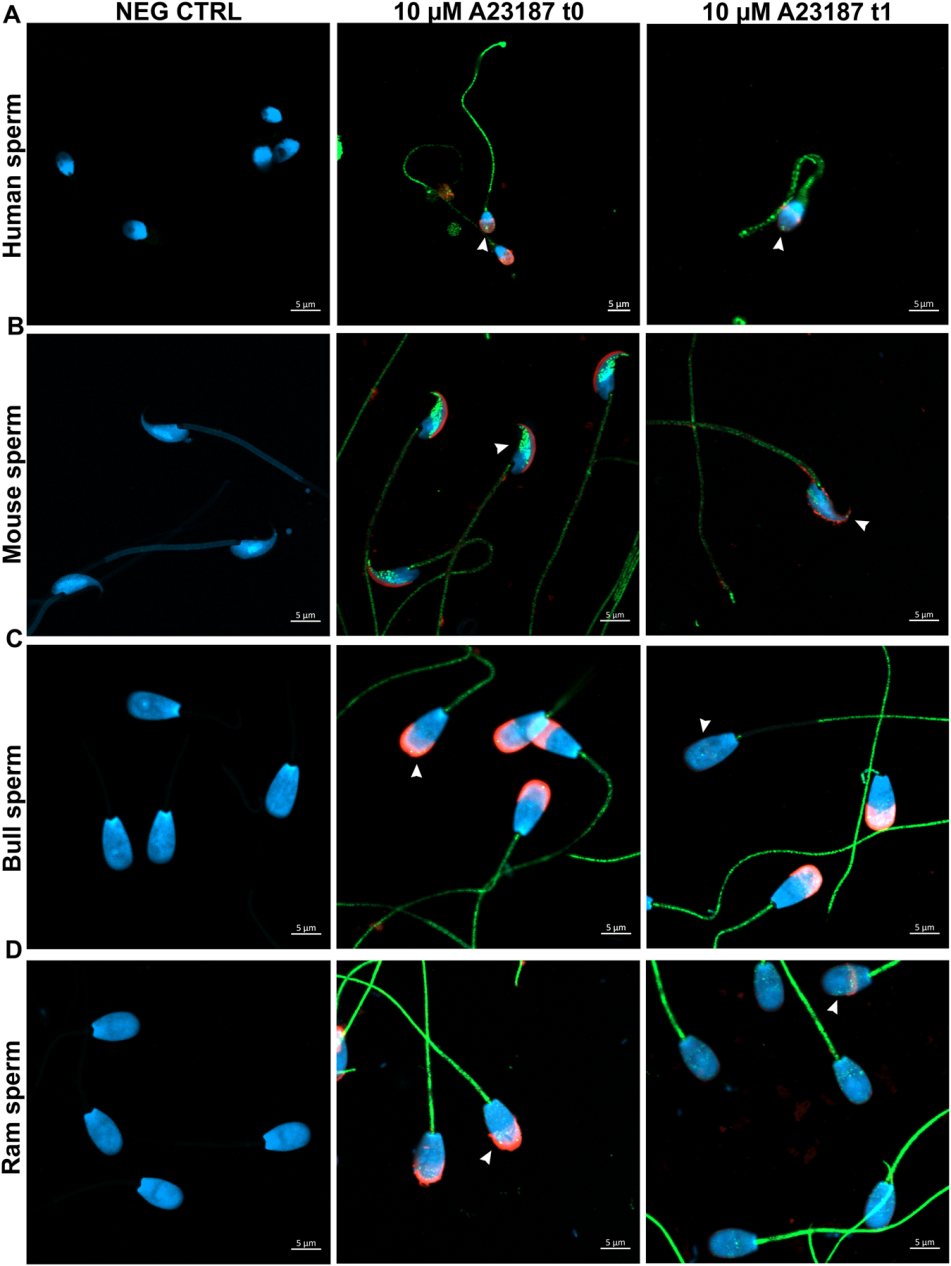
CB1 head spots are present in mammalian sperm before and after the acrosome reaction. Representative confocal images of human (**A**), mouse (**B**), bull (**C**), and ram (**D**) sperm. Negative controls confirm the absence of CB1 signal in sperm processed without the primary antibody. CB1 head spots (green, indicated by white arrowheads) were visible in sperm with an intact acrosome (red) and persist after the acrosome reaction. Nuclei were counterstained with DAPI (blue). Scale bar: 5 µm.

To better understand whether CB1 distribution in the sperm head is conserved across mammalian species and whether CB1 spots persist after the acrosome reaction (indicating localization beneath the plasma and outer acrosomal membranes), immunocytochemistry was performed before and after incubation with the ionophore A23187 (10 µM, 2 h). The results showed that in human sperm, CB1 spots in the acrosomal region remained even after the acrosome reaction (Figs. 3A and S3A Supplementary Materials). In mice, sperm was collected from both the caput and cauda regions of the epididymis. The presence of CB1 spots in the sperm head was notably higher in cauda sperm compared to caput sperm, with the majority localized along the anterior part of the acrosome and fewer in the post-acrosomal region in both cases (Figs. 3B, S2 and S3B Supplementary Materials). Following the acrosome reaction in cauda sperm, CB1 spots remained present, albeit in reduced amounts (Figs. 3B and S3B Supplementary Materials). In bull sperm, CB1 spots exhibited a similar localization before and after the acrosome reaction, being distributed throughout the acrosomal, equatorial, and post-acrosomal regions (Figs. 3C and S3C Supplementary Materials), while in ram sperm CB1 was restricted to the equatorial and post-acrosomal regions before and after acrosome reaction (Figs. 3D and S3D Supplementary Materials).

### Localization of CB1 in non-mammalian sperm

To determine whether the head spot distribution of CB1 is also present in non-mammalian species, immunocytochemistry was performed in two species with an acrosome (sea urchin and rooster), which have different acrosome shapes, compositions, and reactions compared to mammals [34], and in one species lacking an acrosome (zebrafish). The results showed that CB1 was absent in the head of zebrafish (Figs. S4A and S5A, Supplementary Materials) and sea urchin (Figs. S4B and S5B, Supplementary Materials), being present only in the flagellum, where it displayed a spot-like distribution. In contrast, in rooster sperm, CB1 was detected in the acrosomal region but was absent from the rest of the sperm head and the midpiece of the flagellum (Fig. S4C and S5C, Supplementary Materials).

### CB1 functions in the human sperm nucleus

**Fig. 4:**
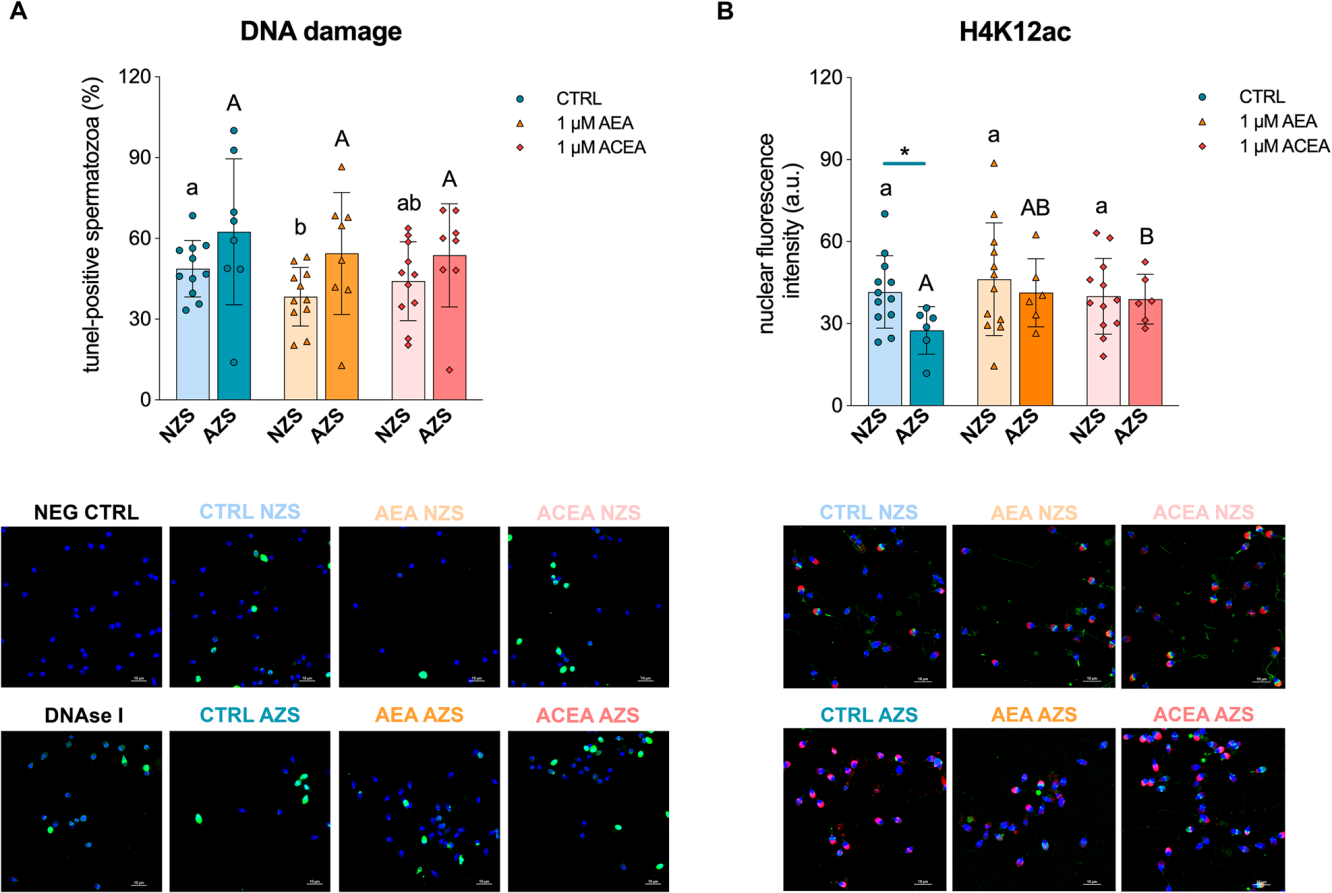
AEA reduces human sperm DNA damage independently of CB1, while CB1 activation regulates H4 acetylation. **A** The percentage of TUNEL-positive cells was similar between normozoospermic (NZS) and asthenozoospermic (AZS) samples. In the NZS group, treatment with 1 µM AEA significantly reduced the percentage of sperm cells with DNA damage compared to the CTRL group (*p* < 0.05, indicated by different lowercase letters). Bars represent mean ± standard deviation (*n* = 11 for NZS, *n* = 8 for AZS). Representative confocal images show the negative control (NEG CTRL, without terminal deoxynucleotidyl transferase), positive control (650 U/mL DNase I-treated sperm), and all experimental groups. TUNEL-positive sperm cells appear green, and nuclei were counterstained with DAPI (blue). Scale bar = 10 µm. **B** Quantification of H4K12ac mean fluorescence intensity in the nucleus revealed significantly lower levels in the CTRL AZS group compared to the CTRL NZS group (*p* < 0.05, indicated by an asterisk). Treatment with 1 µM ACEA significantly increased H4K12ac levels in the AZS group when compared to its own CTRL (*p* < 0.05, indicated by different uppercase letters), increasing acetylation levels to those observed in the NZS group. Bars represent mean ± standard deviation (*n* = 12 for NZS, *n* = 6 for AZS). H4K12ac appears in green, acrosome was stained with Alexa Fluor™ 568-conjugated Lectin PNA (red), and nuclei were counterstained with DAPI (blue). Scale bar = 10 µm.

To investigate the potential role of CB1 in sperm DNA integrity, sperm samples from donors were treated with AEA and ACEA. The samples were classified into two groups based on sperm quality (Table S1 Supplementary Materials): normozoospermic (NZS) and asthenozoospermic (AZS), the latter characterized by reduced sperm motility. The results showed that AEA treatment significantly decreased the percentage of TUNEL-positive cells in the NZS group compared to the control (incubation of sperm cells only with the vehicle, 1% DMSO), while none of the two cannabinoid treatments alter the percentage of sperm cells with DNA damage in the AZS group respect the control (Fig. 4A).

Regarding the involvement of CB1 in H4 acetylation, AZS samples, which exhibited significantly lower H4K12ac levels than NZS samples in the control treatment, showed a marked increase in H4K12ac levels following ACEA treatment, reaching levels comparable to those observed in the NZS group (Fig. 4B).

## Discussion

To date, the localization of CB1 in human sperm has been a subject of debate. Some studies have reported its presence exclusively in the sperm head and midpiece [5, 27], while others have also detected CB1 in the sperm tail [26]. Previous studies relied on widefield fluorescence microscopy, which lacks optical sectioning and is prone to background fluorescence from out-of-focus planes. To overcome these limitations, we used confocal microscopy, which enables optical sectioning and minimizes background fluorescence, providing high-resolution 3D imaging of CB1 distribution within cells [35]. In addition, we used an Airyscan microscope, a super-resolution variant of confocal imaging that enhances lateral resolution through the use of a detector array rather than a single pinhole, allowing more precise localization of receptors within cellular structures [36]. Following immunocytochemistry, confocal microscopy revealed CB1 receptors distributed in a dotted pattern along the human sperm tail, in several midpieces, and as multiple or single larger spots within the sperm head. Notably, Airyscan imaging further demonstrated that a subset of CB1 receptors is localized not only on the sperm surface but also present intracellularly reaching the nuclear area. Besides, the persistence of CB1 signal in human sperm head after ionophore treatment confirms that its localization is not restricted to the plasma membrane or outer acrosomal membrane.

Noteworthy, the intracellular localization of CB1 observed in this study has never been reported in spermatozoa. Instead, such intracellular localization has primarily been reported in cultured cells as Human Embryonic Kidney 293, Chinese Hamster Ovary, and Pituitary corticotrope tumor (AtT-20) cells [37] and primary hippocampal neurons [38]. In these cells, CB1 is known to associate with different adaptor proteins that regulate its endolysosomal targeting and function, including receptor desensitization and the activation of alternative pathways [37]. In addition to endolysosomes, CB1 has been also identified in mitochondria of skeletal and myocardial muscles [39], of astroglia [40], and of spermatozoa [25]. Interestingly, Nielsen et al. [24] reported CB1 localization in the nucleus of human primary spermatocytes. These findings suggest that CB1 has additional roles in human sperm beyond its previously described functions in motility, capacitation, and the acrosome reaction [5, 26]. In this context, CB1 has been shown to affect histone displacement in spermatids by regulating the hyperacetylation of H4 in mice testes, whereas at epididymal level, it regulates mice sperm chromatin condensation by inducing the formation of inter/intra protamine disulfide bonds [23]. In human spermatids, a decrease in H4 hyperacetylation has been reported to hinder histone-to-protamine exchange, thereby impairing spermatogenesis [41]. Therefore, we investigated whether CB1 activation could modulate H4 acetylation on this species. To this end, we conducted in vitro experiments by incubating human spermatozoa from donors with the endocannabinoid AEA and the specific CB1 ligand ACEA. First, our results revealed significantly lower H4K12ac levels in sperm from AZS patients compared to NZS, aligning with previous findings that asthenoteratozoospermic samples (characterized by abnormal motility and morphology) exhibit reduced H4 acetylation, as demonstrated through “bottom-up” nano-liquid chromatography-tandem mass spectrometry [42]. Similar results have been recently published showing that subfertile human sperm display a significantly reduced level of H4K12ac compared to fertile sperm, as evidenced by immunofluorescence and Western blotting [43]. Noteworthy, when we specifically activated CB1 by ACEA treatment, an increase in H4K12ac was shown in AZS patients, increasing its levels up to those observed in the NZS group. To our knowledge, this is the first study to attribute a role to CB1 in human sperm epigenetic remodeling. Interestingly, reduced levels of protamine 2 (PRM2) in subfertile men and *Prm2*-deficient mouse model are associated with lower levels of H4 acetylation in human ejaculated sperm and murine epididymal sperm [43]. Besides, abnormal levels and distribution of H4K12ac characterize human sperm of poor quality [44]. Therefore, the increased levels of H4K12ac after CB1 activation in AZS samples we observed could be explained by incomplete histone–protamine exchange, leaving the retained H4 in mature sperm available for further acetylation. This could have implications also during early embryogenesis, since altered H4K12ac levels within the promoters of developmentally important genes in subfertile men have been associated with insufficient sperm chromatin compaction disrupting the proper transfer of epigenetic marks to the oocyte [45].

Given that the ECS is present in nearly all animals from Cnidaria to mammals, except insects [46], but chromatin sperm compaction mechanisms vary widely, we investigated CB1 localization in sperm from vertebrates (mammals, birds, and fish) and invertebrates (sea urchins) to clarify its evolutionary role. Immunocytochemistry analysis revealed that CB1 was present in the sperm tail of all the species analyzed, while it was detected in the sperm head only in species with internal fertilization: roosters (where it was restricted to the acrosomal region) and all the mammalian species examined. Beyond fertilization strategy, roosters and mammals share a sperm compaction mechanism that involves extensive histone replacement by protamines, whereas zebrafish and sea urchin sperm retain nucleosomal chromatin with histones [29, 47, 48]. As previously mentioned, CB1-mediated H4 acetylation has been shown to influence chromatin condensation in mice by regulating histone removal and promoting protamine disulfide bond formation during epididymal transit, from caput-to-cauda [23]. This underscores the significance of CB1 presence in the mammalian sperm head and explains the differential CB1 content observed in the heads of caput and cauda murine epididymal spermatozoa observed in this study. Nevertheless, CB1 is absent from the sperm head of zebrafish and sea urchins, where this process does not occur. However, since histone-to-protamine transition takes place in rooster sperm, CB1 would also be expected in the sperm head; yet, it is restricted to the acrosomal region. This could be linked to the fact that rooster protamines lack cysteine residues and, consequently, the stabilizing effects of disulfide bonds [47], a process in which CB1 is involved. Moreover, similar to observations in humans, CB1 remained localized in the sperm head of mice, bulls, and rams even after the acrosome reaction. Altogether, these findings suggest that CB1 may play a conserved role in sperm chromatin dynamics across mammalian species.

Beyond histone-to-protamine exchange and sperm epigenetics, CB1 could be also involved in sperm DNA integrity since another study using *Cnr1* knockout mice demonstrated that sperm from both the caput and cauda epididymis exhibited a significantly higher percentage of DNA damage [22]. To investigate the role of CB1 in maintaining human sperm DNA integrity, we treated sperm cells with both AEA and ACEA and we conducted a TUNEL assay. Our results revealed that treatment with AEA significantly reduced the percentage of sperm with DNA fragmentation in NZS samples, whereas ACEA had no such effect. These findings suggest that the observed improvement in sperm DNA integrity is not mediated by CB1 activation. Instead, other endocannabinoid receptors may be responsible for this effect.

In conclusion, our study provides the first evidence of the intracellular localization of CB1 in mammalian sperm head and its involvement in human sperm epigenetic remodeling. Noteworthy, CB1 appears to extend toward the nuclear region, suggesting a potential direct influence on chromatin dynamics. However, the precise intracellular compartmentalization of CB1 within sperm cell and its specific mechanism in modulating histone acetylation remain to be fully characterized. Additionally, while our findings suggest a conserved role for CB1 in mammalian sperm chromatin remodeling, further research is needed to explore its broader function in sperm biology across species.

## Supplementary information

Supplementary Materials

Movie S1

Movie S2

## Data Availability

All data generated or analysed during this study are included in this published article and its supplementary information files.
