# Supplementary material for: From localization to function: comparative analysis of CB1 in sperm across species and its epigenetic role in humans
Source: Cell Death Dis. 2026 Jan 15;17(1):176. doi: 10.1038/s41419-025-08386-2 (PMC12877042; doi:10.1038/s41419-025-08386-2)
Supplement: Supplementary file 1 — Supplementary Materials [file 41419_2025_8386_MOESM1_ESM.docx]

**Title:** From Localization to Function: Comparative Analysis of CB1 in Sperm Across Species and Its Epigenetic Role in Humans

**Runing title:** CB1 Influences Human Sperm Chromatin Remodeling

Marta Lombó^1,2,3^*, Fiorenza Sella^1,3^*, Christian Giommi^1,3^, Stefano Giannubilo^4^, Andrea Frontini^1^, Andrea Ciavattini^4^, Gilda Cobellis^5^, Franceso Manfrevola^5^, Nina Montik^4^, Marina Paolanti^6^, Paz Herráez^2^, Oliana Carnevali^1,3^

^1^Department of Life and Environmental Sciences, Università Politecnica delle Marche, 60131 Ancona, Italy

^2^Department of Molecular Biology, Faculty of Biology and Environmental Sciences, Universidad de León, 24071 León, Spain

^3^INBB—Consorzio Interuniversitario di Biosistemi e Biostrutture, 00136 Roma, Italy

^4^Department of Odontostomatological and Specialized Clinical Sciences, Università Politecnica delle Marche, 60020 Ancona, Italy

^5^Department of Experimental Medicine, University of Campania L. Vanvitelli, Naples 80138, Italy

^6^Department of Political Sciences, Communication and International Relations, University of Macerata, 62100, Macerata, Italy

^*^These authors contributed equally to this work.

Corresponding authors: Oliana Carnevali and Nina Montik **Email:** [o.carnevali@staff.univpm.it](mailto:o.carnevali@staff.univpm.it); [nina.montik@ospedaliriuniti.marche.it](mailto:nina.montik@ospedaliriuniti.marche.it)

**This PDF file includes:**

Figures S1 to S5

Tables S1

Legends for Movies S1 to S2

Supplementary Material References

**Other supporting materials for this manuscript include the following:**

Movies S1 to S2


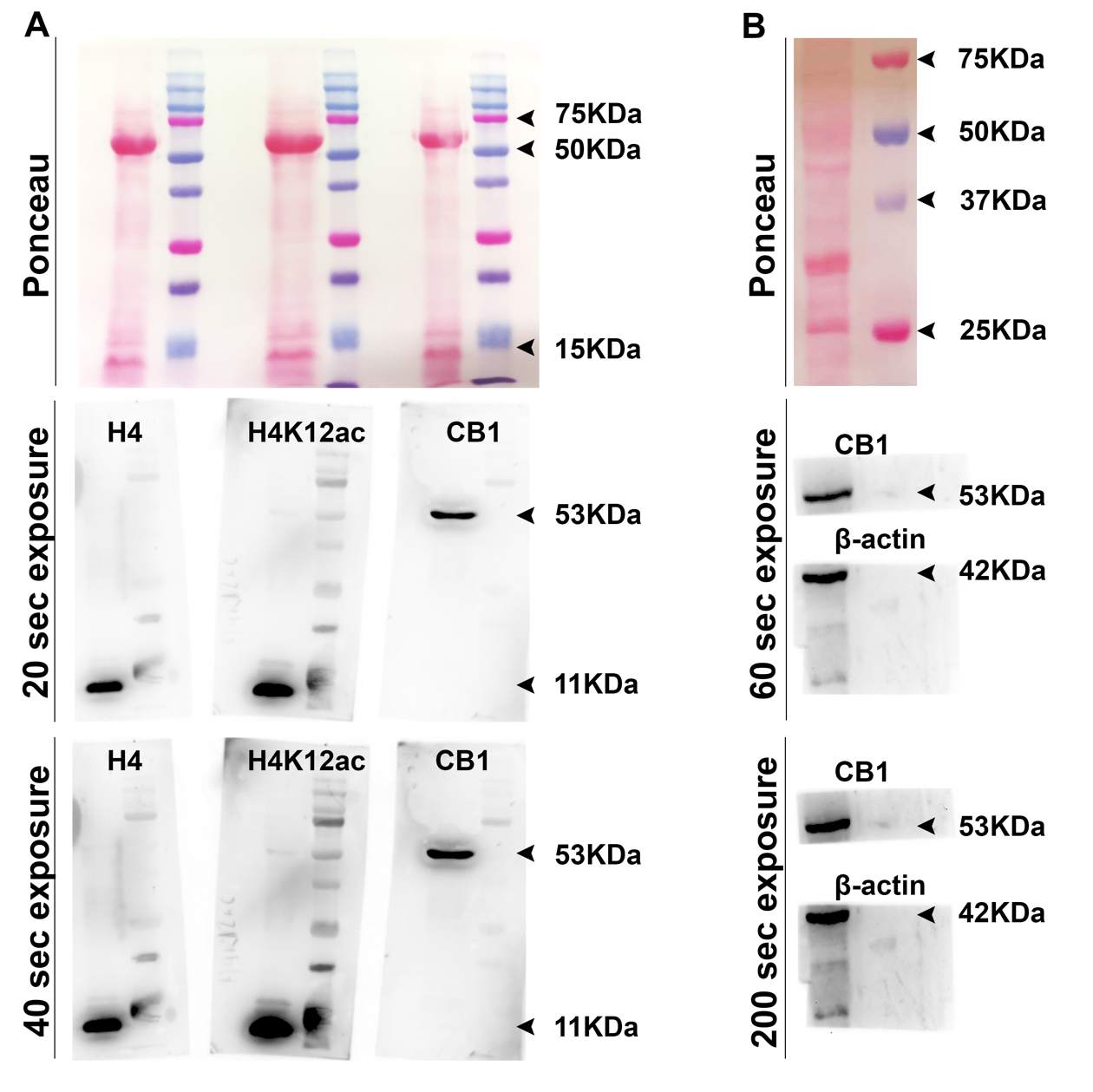


Fig. S1. Detection of CB1 by Western blot in human spermatozoa selected by swim-up. The upper panels show Ponceau-stained SDS-PAGE gels. In human sperm, the lower arrowheads indicate histone reference proteins (H4 and H4K12ac, 11KDa), while the upper arrowheads mark the CB1-specific band at 53 kDa at both 20- and 40-second exposures.


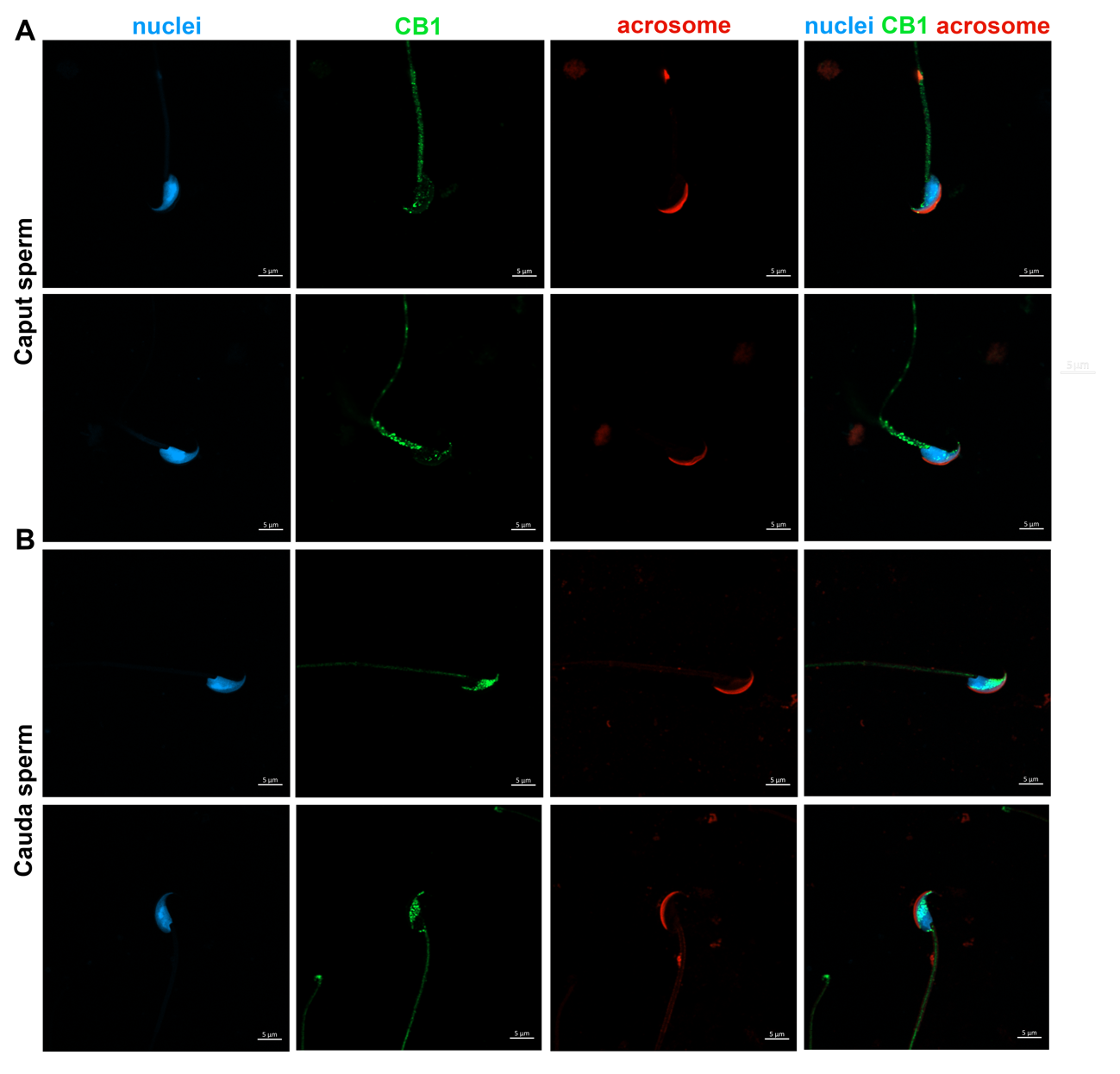
**Fig. S2.** **CB1 spots in the sperm head are localized predominantly in the anterior region of mouse caput and cauda epididymis, with a higher quantity observed in the latter.** Representative confocal images of mouse caput (A) and mouse cauda (B) epidydimal sperm. CB1 spots appear in green, the acrosome was stained with Alexa Fluor™ 568-conjugated Lectin PNA (red), and nuclei were counterstained with DAPI (blue). Scale bar: 5 µm.

**
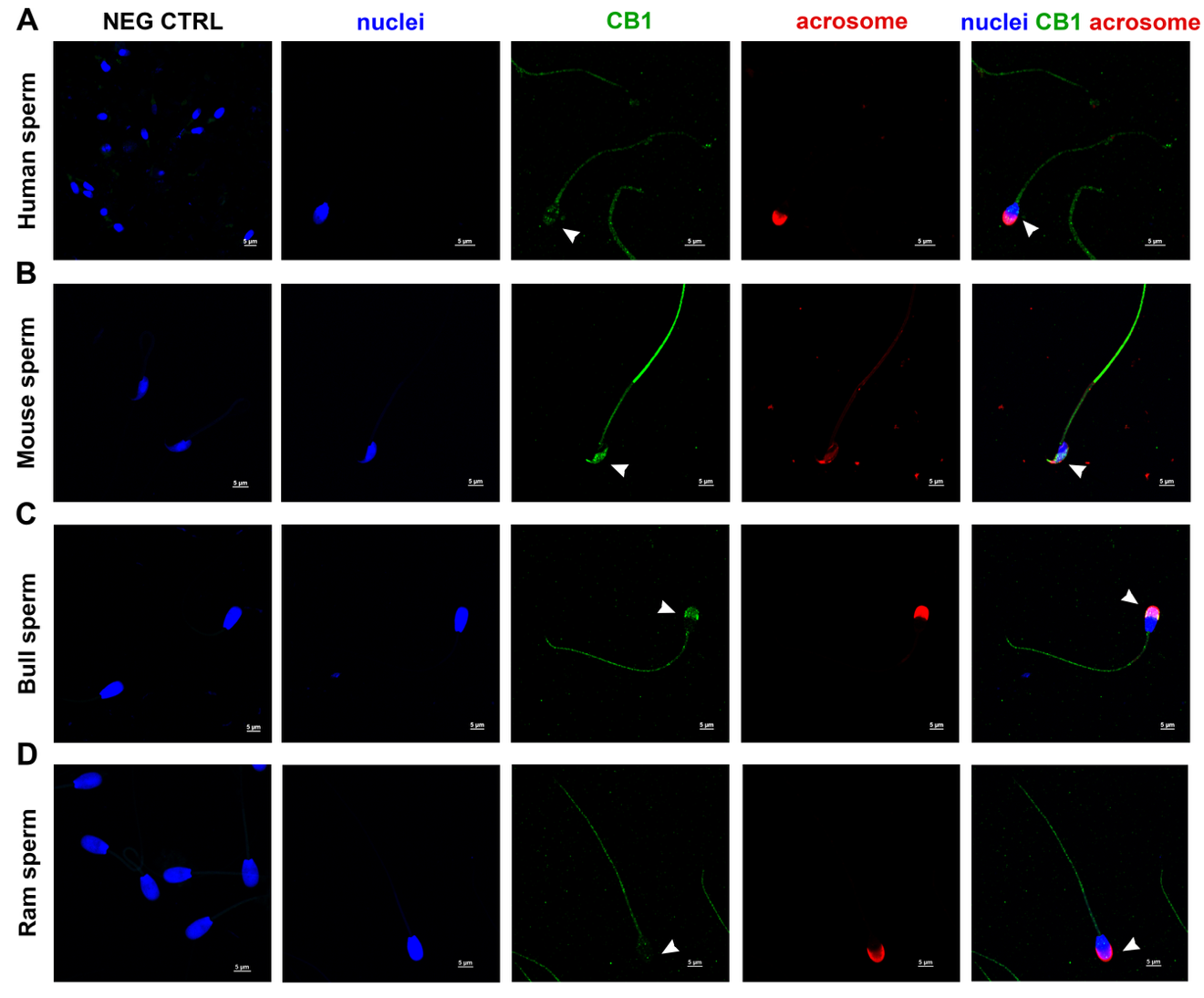
**

**Fig. S3. CB1 head spots are evident in the mammalian sperm head.** Representative confocal images of human (A), mouse (B), bull (C), and ram (D) sperm. Negative controls confirm the absence of CB1 signal in samples processed without the primary antibody, whereas staining with an alternative anti-CB1 polyclonal antibody (ab23703), different from that used in Figure 3, confirms the presence of CB1-positive spots (green, indicated by white arrowheads) in mammalian sperm head before acrosome (red) reaction. Nuclei were counterstained with DAPI (blue). Scale bar: 5 µm.

**
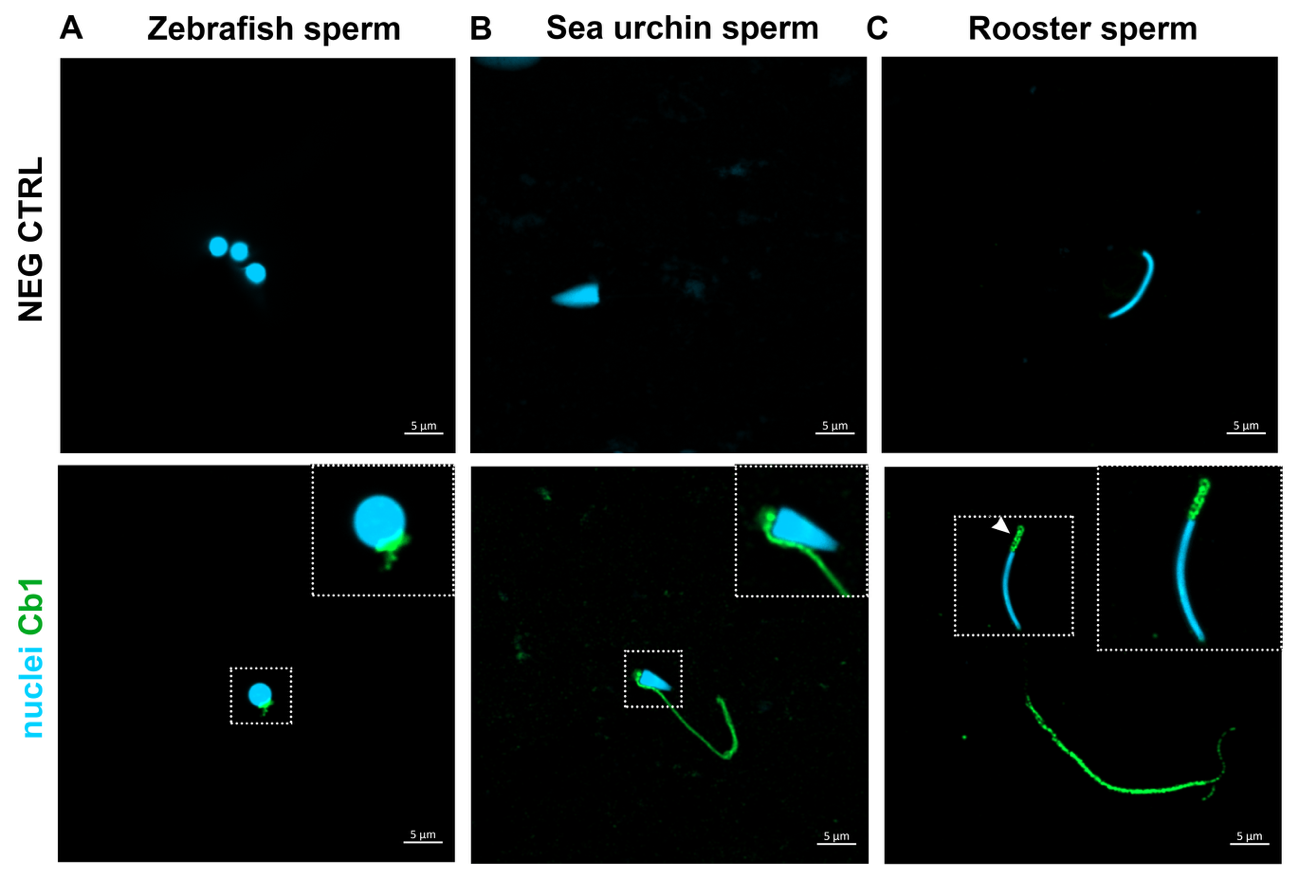
**

**Figure S4. Cb1 is not present in the sperm head of non-mammalian species.** Immunocytochemical analysis revealed that Cb1 (green) is exclusively localized in the flagellum of zebrafish (A) and sea urchin (B) sperm. In contrast, in rooster sperm (C), Cb1 is detected in the anterior portion of the head, corresponding to the acrosome (white arrowhead), as well as in the principal piece and endpiece of the flagellum (with a weaker signal in the latter), while it is absent in the midpiece. High-magnification images (white dashed squares). Negative controls confirm the absence of Cb1 signal in sperm processed without the primary antibody. Nuclei were counterstained with DAPI (blue). Scale bar: 5 µm.

**
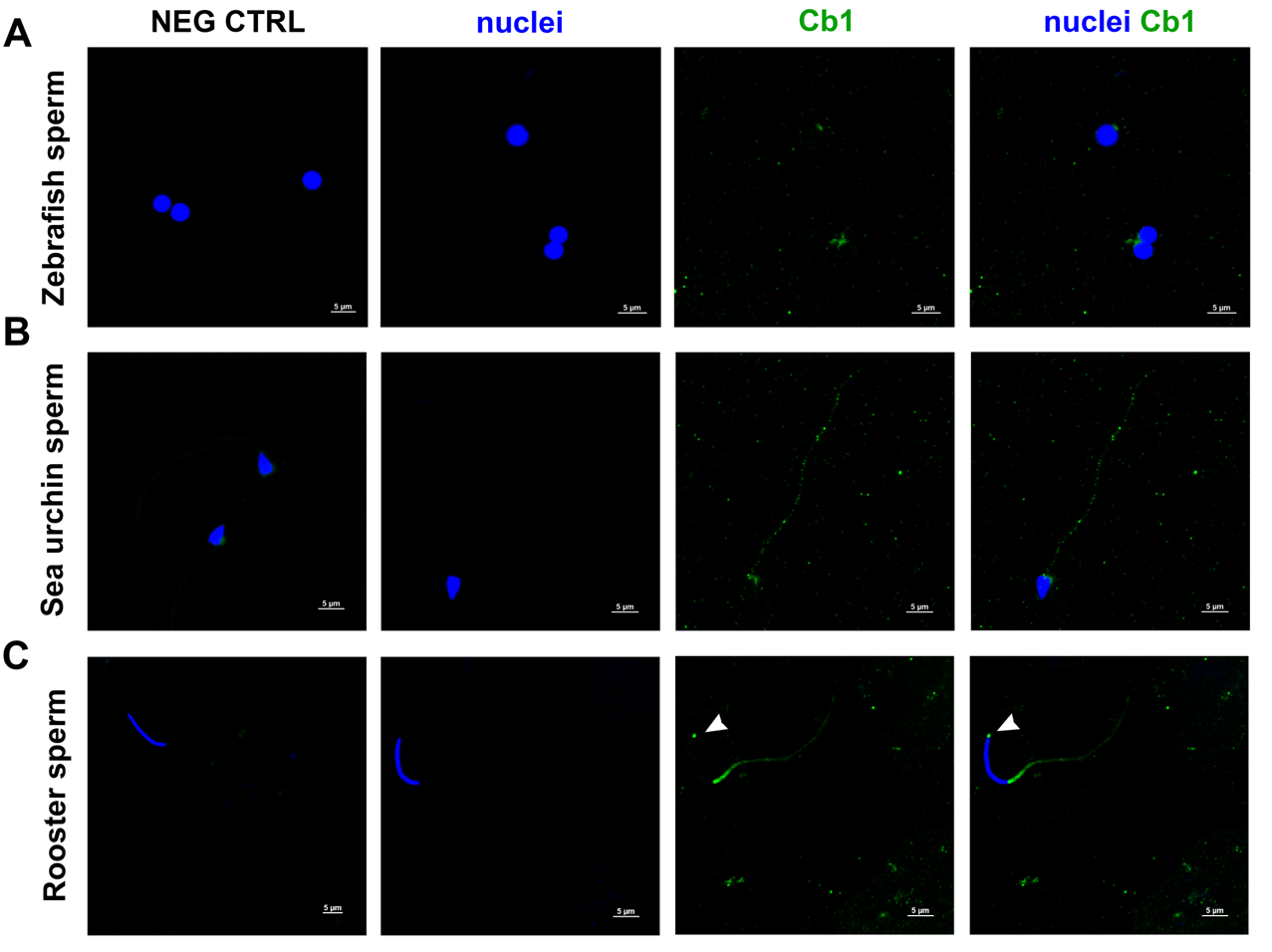
**

**Figure S5. Cb1 in the sperm head of non-mammalian species.** Immunocytochemical analysis using an alternative anti-CB1 antibody (ab23703), different from that used in Figure S4, shows that Cb1 (green) is exclusively localized along the flagellum in zebrafish (A) and sea urchin (B) sperm, whereas in rooster sperm (C) Cb1 is also detected in the anterior region of the head, corresponding to the acrosome (white arrowheads). Negative controls confirm the absence of Cb1 signal in samples processed without the primary antibody. Nuclei were counterstained with DAPI (blue). Scale bar: 5 µm.

**Table S1.** Descriptive features of the 13 normozoospermic (NZS) individuals and the 8 asthenozoospermic (AZS) individuals included in the present study. Spz: Spermatozoa, P: Progressive, NP: Non-progressive, NF: Normal forms. WHO morphology criteria ^1^.

| **Donor** | **Concentration (x10^6^spz/ml)** | **Motility (%P+%NP)** | **Normal Morphology (%NF)** | **Type of donor**  ^1^ |
| --- | --- | --- | --- | --- |
| 1 | 236 | 36 | 7 | ASZ |
| 2 | 31 | 43 | 10 | NZS |
| 3 | 92 | 45 | 18 | NZS |
| 4 | 115 | 54 | 4 | NZS |
| 5 | 35 | 48 | 8 | NZS |
| 6 | 173 | 70 | 30 | NZS |
| 7 | 30 | 28 | 6 | ASZ |
| 8 | 70.1 | 57 | 16 | NZS |
| 9 | 224.2 | 55 | 19 | NZS |
| 10 | 95.8 | 47 | 16 | NZS |
| 11 | 192.3 | 57 | 60 | NZS |
| 12 | 133.4 | 47 | 63 | NZS |
| 13 | 125.3 | 51 | 52 | NZS |
| 14 | 261.6 | 56 | 22 | NZS |
| 15 | 90.4 | 20 | 6 | ASZ |
| 16 | 79.9 | 55 | 12 | NZS |
| 17 | 63.2 | 18 | 9 | ASZ |
| 18 | 204.2 | 40 | 11 | ASZ |
| 19 | 78.4 | 49 | 18 | ASZ |
| 20 | 111.2 | 46 | 14 | ASZ |
| 21 | 20.2 | 36 | 9 | ASZ |

Movie S1. Three-dimensional reconstructions showing rotating views of human sperm cells stained with CB1 (green) and nuclei (blue).

Movie S2. Three-dimensional reconstructions showing rotating views of human sperm cells stained with CB1 (green), acrosome (red), and nuclei (blue). Colocalization of the acrosome and CB1 appears in yellow.

**References**

1 Organization WH. WHO laboratory manual for the examination and processing of human semen. 6^th^ edn. 2021.https://iris.who.int/handle/10665/343208 (accessed 27^th^ November 2025).
